# Current Knowledge on Beetroot Bioactive Compounds: Role of Nitrate and Betalains in Health and Disease

**DOI:** 10.3390/foods10061314

**Published:** 2021-06-07

**Authors:** Iñaki Milton-Laskibar, J. Alfredo Martínez, María P. Portillo

**Affiliations:** 1 Precision Nutrition and Cardiometabolic Health Program, IMDEA—Food Institute (Madrid Institute for Advanced Studies), Campus of International Excellence (CEI) UAM + CSIC, Spanish National Research Council, 28049 Madrid, Spain; jalfredo.martinez@imdea.org; 2 CIBERobn Physiopathology of Obesity and Nutrition, Institute of Health Carlos III (ISCIII), 28029 Madrid, Spain; mariapuy.portillo@ehu.eus; 3 Nutrition and Obesity Group, Department of Nutrition and Food Science, Faculty of Pharmacy and Lucio Lascaray Research Centre, University of the Basque Country (UPV/EHU), 01006 Vitoria-Gasteiz, Spain; 4 BIOARABA Health Research Institute, 01006 Vitoria-Gasteiz, Spain

**Keywords:** beetroot, dietary supplement, nitrate, betalains

## Abstract

An increase in the prevalence of noncommunicable chronic diseases has been occurring in recent decades. Among the deaths resulting from these conditions, cardiovascular diseases (CVD) stand out as the main contributors. In this regard, dietary patterns featuring a high content of vegetables and fruits, such as the Mediterranean and the DASH diets, are considered beneficial, and thus have been extensively studied. This has resulted in growing interest in vegetable-derived ingredients and food-supplements that may have potential therapeutic properties. Among these supplements, beetroot juice, which is obtained from the root vegetable *Beta vulgaris,* has gained much attention. Although a significant part of the interest in beetroot juice is due to its nitrate (NO_3_^−^) content, which has demonstrated bioactivity in the cardiovascular system, other ingredients with potential beneficial properties such as polyphenols, pigments and organic acids are also present. In this context, the aim of this review article is to analyze the current knowledge regarding the benefits related to the consumption of beetroot and derived food-supplements. Therefore, this article focuses on nitrate and betalains, which are considered to be the major bioactive compounds present in beetroot, and thus in the derived dietary supplements.

## 1. Introduction

The prevalence of noncommunicable chronic diseases has been on the rise in recent decades. It is estimated that noncommunicable disease-related deaths account for more than 70% of all deaths that occur worldwide each year [1]. Among them, cardiovascular diseases (CVD), which are strongly related to obesity, stand out as the main contributors. Indeed, obesity is considered a major risk factor for the development of arterial hypertension, myocardial infarction or stroke, as well as other chronic metabolic disorders (diabetes and dyslipidemia) that may have an influence on CVD mortality [1]. In this line, the benefits of dietary patterns characterized by a high intake of vegetables and fruits in CVD have been extensively studied. The Mediterranean and the Dietary Approaches to Stop Hypertension (DASH) diets are the most widely studied ones [2,3,4,5]. This relationship between vegetable-rich diets and CVD has resulted in growing interest in vegetable-derived ingredients and plant-food based supplements that may have potential therapeutic properties [6].

One of these food supplements is beetroot juice, which is obtained from the root vegetable *Beta vulgaris*. Much of the interest in beetroot juice derives from its nitrate (NO_3_^−^) content, which is known to have bioactivity in the cardiovascular system [7]. However, beetroot juice also contains further ingredients such as polyphenols, pigments and organic acids that may well be of benefit [6]. Nitrate has also been reported to improve performance in sport, and this has led to the use of beetroot juice as a common food supplement as an ergogenic aid [8]. The aforementioned effects attributed to beetroot juice have contributed to an increase in its popularity, and therefore, in its consumption. It has been reported that the global market of beetroot juice increases by 5% every year, a trend that is estimated to remain steady in the following years [9].

In this scenario, the aim of this review article is to analyze the current knowledge regarding the benefits related to the consumption of beetroot and derived food-supplements or formulations, as well as the involved mechanisms of action. To this end, the present article focuses on nitrate and betalains, which are considered to be the major bioactive compounds present in beetroot, and thus, also in the derived dietary supplements.

## 2. Nitrate

**Figure 1:**
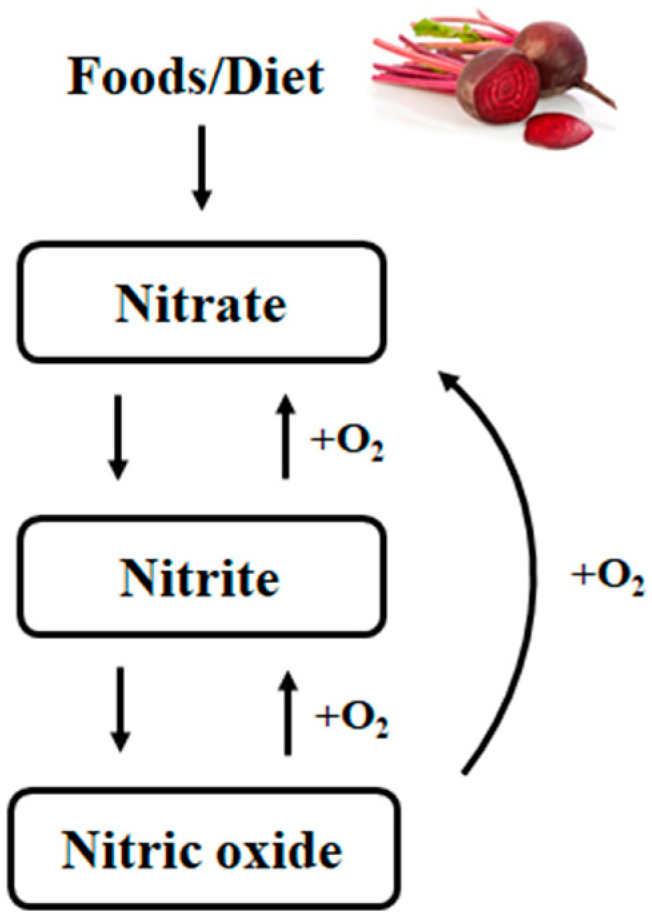
Nitrate–nitrite–nitric oxide pathway. Adapted from Niayakiru et al., 2020 [11].

The main sources of nitrate in mammals are diet (especially leafy green vegetables) and endogenous synthesis (the majority of cells in our body can synthetize nitrate) [10]. At first, nitrate, as well as nitrite (NO_2_^−^), were thought to be an inert derivative of the metabolism of nitric oxide (NO). Nowadays, it is well-known that both nitrate and nitrite can be NO precursors (Figure 1) through the nitrate–nitrite–NO synthase pathway [11]. This conversion mainly takes place in conditions of hypoxia and/or acidosis, as occurs in muscle contraction or tissue ischemia [12]. It has also been described that this metabolic pathway is triggered when nitrate comes from dietary sources, i.e., after consuming leafy green vegetables [13].

As stated before, the main dietary sources of nitrate are leafy green vegetables (lettuce, spinach or arugula) and beetroot, providing 50–85% of the total dietary nitrate intake [7]. Nitrate is also present in fruits and fruit juices, being especially abundant in beetroot juice. As far as foods and foodstuffs of animal origin are concerned, since nitrate is commonly used to preserve foods such as sausages, cold meat and cured meat (as nitrite), it will also be present in these products [14].

The nitrate content in different vegetables and fruits has already been reported (Table 1). However, the array of factors that are known to affect this parameter makes it difficult to provide accurate information [15]. For instance, the growing conditions (i.e., the fertilizers used and/or light exposure), as well as the processing of the vegetables (storage, cleaning, peeling or boiling), are some of the factors that may affect the nitrate content. Additionally, the nitrate content in a given vegetable is not homogeneous. Therefore, if some parts are discarded (such as the leaves), the amount of consumed nitrate will be reduced [15].

Interestingly, the intake of foods that are rich in nitrate differs among countries, as well as among regions within the same country, and therefore, may influence the average nitrate intake of a given individual [15]. It is estimated that the average nitrate intake in the United States is of 40–100 mg/day, while in Europe it is of 50–180 mg/day [15]. According to data reported in different studies, nitrate intake tends to be lower in European countries of northern latitudes such as Norway (31 mg/day), Sweden (48 mg/day) or Denmark (50 mg/day), while it is higher in more southern countries such as France (121 mg/day). In all of these cases, the majority of nitrate (80–85% of the total) is provided by vegetables [16]. In addition, other authors have suggested that in vegetarians, a two to four fold increase in nitrate intake may occur in comparison to subjects not following such diets [17]. As far as the Mediterranean countries are concerned, the nitrate intake through fruits and vegetables is thought to be higher due to the composition of the Mediterranean diet [15].

### 2.1. Bioavailability

Once nitrate-rich foods (such as beetroot juice) are consumed and the nitrate is absorbed, plasma levels increase. Then, salivary glands uptake 25% of the nitrate present in the circulation while the rest undergoes renal excretion [18]. The nitrate absorbed by salivary glands is concentrated and excreted into the saliva. Subsequently, it is reduced to nitrite by the commensal bacteria present in the dorsal side of the tongue [19]. The nitrite is then swallowed and absorbed in the gastrointestinal tract, reaching circulation. Finally it is transported to different locations within the body where it can be further reduced in order to synthesize NO or other compounds [11]. The existence of this enterosalivary pathway has been demonstrated in studies in which patients were asked not to swallow saliva after having ingested nitrate. Under these conditions, it was observed that the characteristic increase of nitrate levels in the blood did not occur [20]. In addition, studies conducted in rodent models and humans have demonstrated that as well as in blood, nitrate also occurs in the liver and muscle [21]. Indeed, it has been reported that the basal levels of both nitrate and nitrite in skeletal muscle are higher than those found in plasma [22]. Similarly, it has been observed that after beetroot ingestion, nitrate and nitrite contents are increased in plasma and muscle. Therefore, it has been suggested that muscle may act as a reservoir of dietary and endogenous nitrate [23].

According to the available data, the ingestion of dietary nitrate produces an increase in nitrate and nitrite levels in blood that can last up to 24 h. In these conditions, it is believed that NO availability is increased through the nitrate–nitrite–NO synthase pathway. In addition, studies conducted in humans revealed that after consuming either nitrate supplements or a diet containing nitrate-rich foods (leafy green vegetables), plasma nitrate and nitrite increased [13].

### 2.2. Beneficial Health Effects of Nitrate

#### 2.2.1. Antihypertensive Effects

The administration/consumption of nitrate for the prevention and/or management of CVD is based on its capacity to be converted into NO, which is known to regulate blood flux (Table 2). Thus, in young subjects, the supplementation of nitrate salts or beetroot juice (in dietary doses of nitrate) effectively reduces systolic and/or diastolic arterial pressure [24,25,26]. Moreover, the effect of the intake of a 500 mL dose of beetroot juice (containing 1400 mg of nitrate) on arterial tension is similar to that produced by antihypertensive drugs. Interestingly, these effects are known to be present for up to 24 h after beetroot juice ingestion [27]. In general terms, it has been suggested that the effect exerted by nitrate supplementation on arterial pressure is more marked in subjects that exhibit greater impairments of this parameter at baseline [28]. In addition, the age of the subjects seems to affect the outcome of nitrate supplementation concerning arterial pressure, being greater in younger subjects. In this scenario, changes in the oral microbiota and the lowered acid production in the stomach in older subjects may result in a decreased conversion of nitrate into nitrite and nitrite into NO; these changes may explain the lower response identified in these subjects [28]. However, there are also studies showing that the supplementation of 140 mL/day of beetroot juice (≈595 mg nitrate/day) for three days resulted in a significant decrease in systolic and diastolic arterial pressures in subjects whose age ranged from 60 to 70 years [29].

With regard to the effect of dietary nitrate on arterial pressure, the evidence is not as clear as that for supplements. Traditionally, diets rich in vegetables (such as the DASH diet) have been considered beneficial in reducing arterial pressure [3,30]. However, the antihypertensive effects that these diets may exert cannot be solely attributed to their nitrate contribution [4]. Thus, the macronutrient composition, as well as the fiber and antioxidant content found in these dietary patterns, may well influence the improvement in arterial pressure. Additionally, the growing conditions and the time of the year in which vegetables are harvested can also influence their nitrate content. Consequently, at present, there is not enough scientific evidence to state that the dietary intake of nitrate can reduce the risk of CVDs [4].

#### 2.2.2. Effects on Cognitive Function

One of the mechanisms involved in the deterioration of cognitive function is cerebral hypoperfusion. This results in reduced blood flux in the brain that is related to NO activity impairment [31]. According to the available literature, a diet rich in nitrate (including beetroot juice) effectively increases regional cerebral blood flux (mainly in the frontal cortex) in older adults (≈75 years) [32]. Similar results have also been reported in studies in which acute doses of beetroot juice (450 or 500 mL, providing 342 or 750 mg of nitrate, respectively) were given to healthy young adults [33,34]. Interestingly, in one such study, an increase in prefrontal cortex perfusion was described when subjects receiving the beetroot juice were performing cognitive tasks [33].

These data suggest that the consumption of nitrate-rich foods may be a useful approach to enhance the blood flux in specific cerebral areas that control executive function. However, more studies are warranted in order to elucidate whether the increase in regional cerebral blood flux is indeed accompanied by improvements in cognitive function [35].

#### 2.2.3. Nitrate as an Ergogenic Aid to Improve Exercise Performance

Nitrate supplements (salts and beetroot juice) can produce NO enhancement. This, in turn, affects muscle function, resulting in the improvement of exercise performance [36]. Remarkably, when nitrate is consumed along with polyphenols (e.g., when beetroot juice is consumed), the effects on exercise performance are enhanced. It has been proposed that polyphenols may protect nitrate (as well as the derived NO) from the damage induced by reactive oxygen species, thus enhancing its bioavailability [37]. This fact would explain the greater effects observed in exercise performance when consuming beetroot juice, compared to that achieved by the consumption of an equivalent dose of sodium nitrate (NaNO_3_). Interestingly, the effects induced by beetroot/nitrate supplementation seem to be greater in untrained subjects than in athletes [37].

Different studies have reported that acute or chronic supplementation of beetroot juice results in decreased oxygen demands during exercise. Therefore, several beneficial effects have been described regarding the parameters related to cardiovascular and respiratory systems. Interestingly, improvements in economy (greater power generated or distance travelled with the same oxygen consumption), maximum strength, time-to-exhaustion and time trials have been reported [13]. These effects have been described both in trained subjects (cyclists, swimmers and athletes) and untrained ones, as well as under different supplementation protocols. For instance, the observations mentioned above were made in protocols where participants ingested significantly different amounts of beetroot juice (70 to 500 mL), resulting in different nitrate intakes (300–600 mg/day). Moreover, the aforementioned effects were observed under acute (2–3 h) and chronic (3–15 days) supplementation protocols [36]. It should be noted that some studies have suggested that the effects described for beetroot juice supplementation may be achieved by the consumption of a diet which is rich in nitrate. In this sense, the improvements in different parameters related to exercise performance (reduced oxygen consumption during aerobic bike exercise, higher muscle work and strength peak) were described in cyclists who consumed a diet rich in nitrate (8.6 mmol/day) compared to cyclists on a control diet (2.9 mmol/day) [38].

Finally, there is no evidence to support that beetroot juice/nitrate supplementation improves exercise performance under conditions of hypoxia [36].

### 2.3. Interactions and Adverse Effects of Nitrate Consumption

Several dietary components, as well as nondietetic agents, have been shown to interact with nitrate and/or nitrite, interfering with their antihypertensive effects. One such compound is thiocyanate, which competes with nitrate to be absorbed by salivary glands [39]. Higher levels of thiocyanates in the blood have been related to the consumption of vegetables from the Brassica family (cauliflower, cabbage, broccoli, etc.). Cigarette smoke may also impair the metabolism of nitrate, and hence, blunt its effects on arterial pressure. By contrast, it has been described that the consumption of foods and foodstuffs rich in polyphenols (such as berries, nuts or wine) can enhance nitrite reduction in the stomach, which, in turn, may boost the effect of nitrate on arterial pressure [39]. Similarly, different studies have demonstrated that ascorbate can influence the nitrite-derived NO production. Actually, low doses of ascorbate can increase gastric NO production and thus enhance the antihypertensive effect of nitrate supplementation. In contrast, high ascorbate doses may impair NO synthesis, thus reducing the beneficial effects of nitrate supplementation on arterial pressure [39].

Several drugs such as gastric acid-suppressing agents (proton pump inhibitors) or drugs developed to treat gout (inhibitors of xanthine oxidoreductase) have also been linked with lower nitrate-derived NO availability [39]. In the case of gastric acid-suppressing agents, the reduction produced in gastric secretion decreases the NO activation mediated by chlorhydric acid. Indeed, it has been reported that the antihypertensive effect of nitrate supplementation disappears in healthy subjects under this kind of treatment. Regarding the drugs used to treat gout, it must be taken into account that their mechanism of action is based on xanthine oxidoreductase inhibition. This enzyme converts the nitrite that has not been reduced in the stomach into NO; therefore, its inhibition has been related to a lower production of NO [39].

Additionally, antiseptic mouthwashes may eliminate up to 94% of the oral commensal bacteria that reduces nitrate to nitrite. Indeed, clinical trials have demonstrated that the effects of antihypertensive drugs are diminished or even inhibited (totally or partially) in subjects using this type of mouthwash [39].

**Figure 2:**
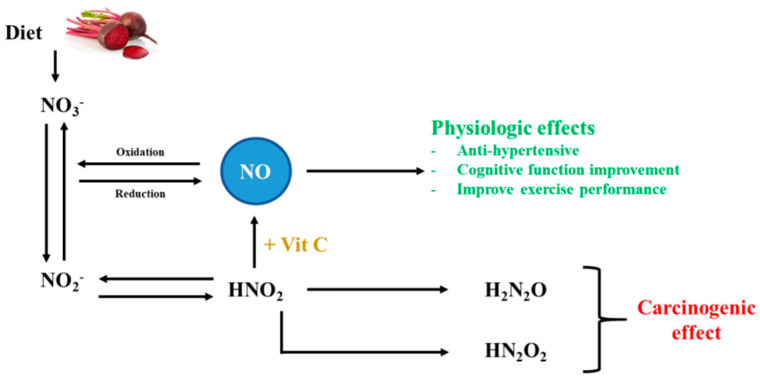
Conversion of nitrate and nitrite into nitrosamines. NO_3_^−^: nitrate, NO_2_^−^: nitrite, NO: nitric oxide, HNO_2_: nitrous acid, H_2_N_2_O: nitrosamines, HN_2_O_2_: nitrosamides, Vit C: vitamin C. Adapted from Berends et al., 2019 [40].

Another issue that has gained much attention is the potential formation of nitrosamines, which have carcinogenic potential, as a result of nitrate consumption (from dietary sources or supplementation). When nitrite is ingested (directly or as nitrate), it can be converted into nitrosating agents in the stomach. This conversion is mediated by the effect of chlorhydric acid and bacteria-mediated enzymatic activities. It is worthy of note that nitrosating agents and nitrosamine can also form from NO due to autoxidation, as well as due to the activity of different NO synthases (Figure 2) [15].

Taking into account the risk associated to nitrosamine formation, the potential relationship between dietary nitrate ingestion and the development of certain types of cancer (stomach and bladder cancer among them) has been widely investigated [7]. Many of these studies have been focused on the relationship between the consumption of red meat and processed meat and cancer development. According to the available data, there is no scientific evidence to support the hypothesis that the consumption of nitrate from dietary sources/drinking water increases the risk of developing cancer [7]. In the case of supplements, a recent study has found enhanced levels of nitrosating agents with carcinogenic potential in athletes supplemented with beetroot juice (providing 400 mg of nitrate) [40]. Nevertheless, studies conducted to date in humans addressing the potential effect of long-term nitrate supplementation on the development of cancer are scarce and inconclusive.

Remarkably, further metabolic and endocrine adverse effects due to nitrate consumption have been described. In this line, nitrate negatively affects thyroid function, since nitrate anions and iodine compete for thyroid gland uptake. In addition, several authors have indicated that nitrate may inhibit steroid hormone synthesis due to the binding of nitrite-derived NO with the hemoglobin in cytochrome P450 enzymes [7].

Finally, it must be noted that few studies have addressed the effect of chronic high-intake of nitrate on arterial pressure. Despite the fact that a diet rich in vegetables is considered to be beneficial for arterial pressure, this effect cannot be attributed solely to nitrate consumption. Studies conducted in animals have demonstrated that the effect of nitrate consumption in arterial pressure tends to diminish when the treatment is maintained over time [41]. Therefore, further studies in humans are necessary to determine whether the effects observed in animals also occur in human beings.

## 3. Betalains

**Figure 3:**
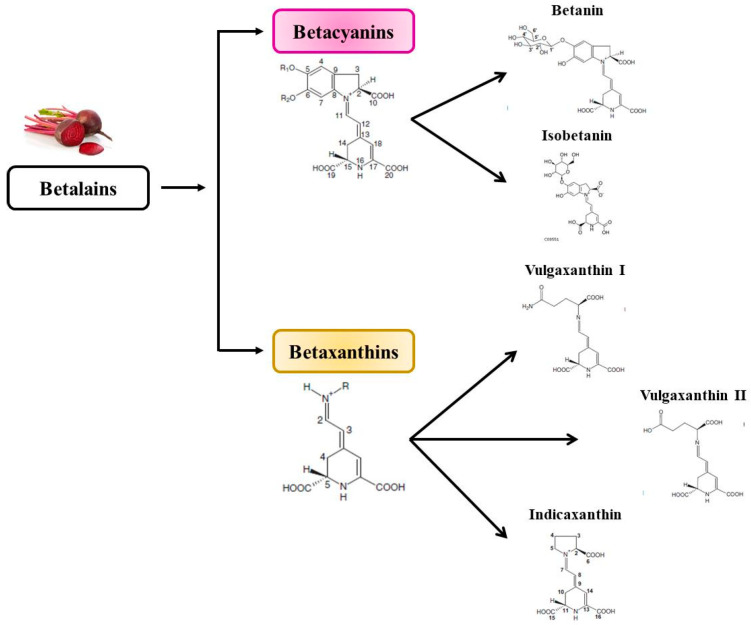
Classification of the two main groups of betalains present in beetroot. All the chemical structures included in this figure were obtained from Khan et al., 2015 [46].

Along with nitrate, betalains are another class of major bioactive compounds which are naturally present in beetroot. Betalains are hydrophilic nitrogenous pigments that are widely used in the food industry as natural colorants for products such as processed meat, ice creams or baked goods [42]. Betalains are classified into two main groups according to their color: betacyanins have a red-violet color, while betaxanthins are yellow-orange. With regard to their chemical structure, betacyanins are made up of betalamic acid linked to a *cyclo*-3,4-dihydroxyphenylalanine. By contrast, betaxanthins feature an amino acid or amine linked to betalamic acid [43]. Although the presence of betalains is limited in vegetables, prickly pear (*Opuntia ficus-indica*), amaranthus, pitaya (*Hylocereus undatus*) and strawberry blite have been shown to contain significant amounts [44]. Among the different pigments categorized as beetroot betalains (Figure 3), betanin (a betacyanin) is the most abundant. Nevertheless, the betalain content may vary depending on factors such as beetroot variety, growing and postharvest storage conditions. In this regard, the usual betacyanin:betaxanthin content of beetroot is 1:3, while the average betalain content in beetroot is 120 mg/100 g of fresh weight [45]. Moreover, betalain content also varies within the beetroot itself, with the peel containing the most [43].

Much attention has been paid to betalains in recent years due to their antioxidant capacity [43]. However, since crude betalain extracts may also contain further compounds with potential bioactive activity (e.g., phenolic acids), much care is needed when interpreting the beneficial effects of these kinds of preparations [42].

### 3.1. Bioavailability

Oral bioavailability of betalains is considered to be relatively low, representing an important issue with regard to their potential health effects. Since betalains are not metabolized by hepatocytes, these pigments reach the systemic circulation without structural modifications [47]. In vitro studies designed to mimic the human gastrointestinal tract (Caco-2 cell monolayer) have demonstrated that betalains are absorbed in their unchanged form through paracellular absorption. This suggests that their molecular activity may be maintained when reaching the systemic circulation [48]. Betalains are also detected in this form in urine, and thus, this approach has been used to investigate the bioavailability of these compounds [47]. According to previous studies, ≈0.3–0.9% of ingested betalains (after consumption of 300–500 mL beetroot juice) are eliminated in the first 12–24 h after the ingestion of the bolus [49,50]. Taking into account that the renal excretion of betalains is low, the existence of other pathways that may contribute to their elimination (such as biliary excretion, bacterial degradation or enterohepatic metabolism) cannot be ruled out [50]. Moreover, further investigations are required to elucidate whether betalains undergo structural transformation which results in the formation of secondary metabolites [6].

### 3.2. Beneficial Health Effects of Betalains

#### 3.2.1. Effects on CVD

Although much of the interest in the use of beetroot juice for the prevention of CVD is due to its nitrate content and antihypertensive effects, some studies have addressed the potential use of betalain-rich beetroot supplements to treat CVD. It has been reported that a two-week regime of a betalain-rich extract (50 mg/day) decreased the levels of total cholesterol (TC), triglycerides and non-high-density lipoprotein cholesterol (non-HDL-c), as well as TC/HDL-c and low-density lipoprotein cholesterol (LDL-c)/HDL-c ratios in the plasma of nonsmoking adults with coronary artery disease [51]. Moreover, significant reductions in systolic and diastolic blood pressure were also observed in this study in subjects treated with the betalain-rich supplement [51]. The authors suggested that the antihypertensive effect exerted by the tested betalain-rich beetroot supplement may have been mediated by the reductions observed in plasma homocysteine levels [51]. In this respect, a positive association between plasma homocysteine levels and hypertension was reported. Thus, elevated homocysteine levels can lead to endothelial cell damage, a reduction in vessel flexibility and impairment in vasodilator factor synthesis, resulting in hypertension [52].

Despite the fact that the aforementioned observations could be considered promising for the usage of betalains/betalain-rich supplements as antihypertensive agents, further studies are warranted. Furthermore, additional or synergic antihypertensive effects of nitrate and betalains cannot be ruled out, since both compounds can be present in the same product, as occurs with beetroot juice.

#### 3.2.2. Antioxidant Effects

Betalains, due to their ability to scavenge reactive oxygen species, as well as to induce antioxidant defense, are well-known antioxidants [53]. According to data obtained in in vivo studies, betalains proved to be effective at protecting against the oxidation of LDL molecules due to their radical-scavenging activity [47,54]. Similar results were reported in humans when the effect of a beetroot-derived, solid, betalain-rich formulation was tested for three consecutive days at a dose of 90 mg of betalain/day. In this case, not only reduced LDL-c oxidation and incremented HDL-c/LDL-c ratio values were reported, but also an increased inhibition of nuclear factor κB (NF-κB), which is a well-known oxidative stress responsive transcriptional factor [55]. In addition, it has been suggested that the protective effect of LDL molecules described for betalains may also be mediated through the activation of the hepatic antioxidant enzyme paraoxonase 1 [56].

Besides the aforementioned observations, hepatoprotective effects have also been attributed to betalains and betalain-rich beetroot preparations, which occur through antioxidant mechanisms. For instance, the toxic effect of 7,12-dimethylbenz(a)anthracene (DMBA) in the liver of female Sprague-Dawley rats was alleviated by the administration of 500–600 mL/day of a betalain-rich beetroot juice (providing 79.3 mg betaxanthins and 159.6 mg per 100 mL juice). This intervention improved the overall antioxidant status and enhanced the expression of quinone oxidoreductase [57]. Moreover, improved hepatic redox status and enhanced mitochondrial function were also described in a similar animal model (male Sprague-Dawley rats) that underwent hepatic injury by paraquat administration and received a betanin extract containing 25 or 100 mg betanins for five days (three days before and two days after paraquat administration) [58]. Similarly, reduced lipid peroxidation was observed in rodents exposed to several toxicants (paraquat, paracetamol, diclofenate and carbon tetrachloride) and then treated with betanins, as suggested by the lowered malondialdehyde (MDA) levels found in liver and kidney [59,60,61].

**Figure 4:**
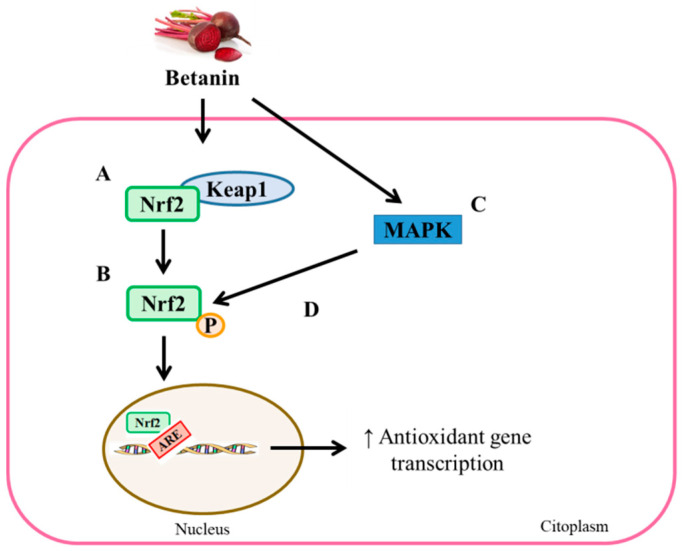
Antioxidant effects induced by betanins through Nrf2. Betanins enhance the Nrf2-Keap1 complex dissociation (**A**) and enhance Nrf2 nuclear translocation (**B**). Additionally, betanins can also activate MAPK (**C**), which, in turn, phosphorylates and stabilizes Nrf2 (**D**), contributing to its nuclear translocation. As a result, nuclear Nrf2-ARE binding is increased, resulting in enhanced transcription of genes encoding antioxidant and phase II enzymes. ARE: antioxidant response element, Keap1: Kelch-like repressor protein ECH-associated protein 1, MAPK: mitogen-activated protein kinase and Nrf2: nuclear factor erythroid 2-related factor 2.

Some of the antioxidant effects described for betalains are mediated by their capacity to modulate the expression and activity of different antioxidant enzymes. Thus, betanins have the ability to increase the expression of nuclear factor erythroid 2-related factor 2 (Nrf2), as well as its levels in the nucleus. In addition, betanins also enhance the binding of Nrf2 and antioxidant response element (ARE) sequences, resulting in the upregulation of genes encoding antioxidant and phase II enzymes [62]. Interestingly, it has been proposed that betanin-mediated Nrf2-ARE binding may result from the modifications induced by betanins in different cysteine residues of Kelch-like repressor protein ECH-associated protein 1 (Keap1), leading to Nrf2-Keap1 complex dissociation. Moreover, betanins are also thought to be involved in mitogen-activated protein kinase (MAPK) activation. This results in the phosphorylation and stabilization of Nrf2, thereby enhancing its nuclear translocation (Figure 4) [62].

In summary, the available data supports the antioxidant capacity attributed to betalains (especially betanin). Current knowledge highlights the ability of these compounds to not only scavenge or suppress oxidant production, but also to modulate the endogenous antioxidant system. In addition, it must be noted that depending on the preparation employed (beetroot juice or betalain extracts), additional compounds (mainly phenolic compounds) may also contribute to the total antioxidant effect.

#### 3.2.3. Anti-Inflammatory Effects

Anti-inflammatory capacity has also been reported for betalains [63]. According to data obtained in in vitro studies, betalains were shown to effectively repress intercellular cell adhesion molecule-1 (ICAM-1) in endothelial cells treated with cytokines [64]. Moreover, betalains have also demonstrated the ability to inhibit lipoxygenase (LOX) and cyclooxygenase (COX) enzymes. These enzymes are known to mediate the conversion of arachidonic acid in pro-inflammatory mediators [65]. In this line, in vivo studies have revealed that these effects occur through the interactions of betalains with different serine and tyrosine residues (in the case of COX), or with substrate-binding amino acids (in the case of LOX) [66].

**Figure 5:**
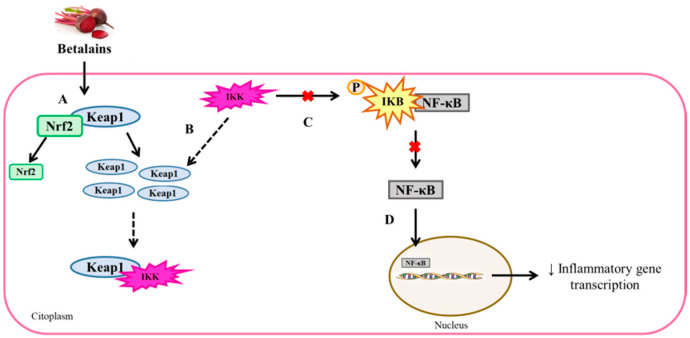
Anti-inflammatory effects of betanins through the modulation of Nrf2-Keap1 and NF-κB-IKB complex dissociation. IKB: KB inhibitor, IKK: ikB kinase, Keap1: Kelch-like repressor protein ECH-associated protein 1, NF-κB: nuclear factor κB and Nrf2: nuclear factor erythroid 2-related factor 2.

In addition, some of the anti-inflammatory properties attributed to betalains derive from their ability to interact with NF-κB, which plays a major role in the activation and transcription of gene targets that regulate the inflammatory response. In this respect, betalains have the ability to suppress NF-κB binding with DNA, and thus, to downregulate the transcription of inflammatory cytokines. This effect has been reported in different in vivo studies. In one such study, the administration of betanin extracts (25 or 100 mg/kg/day for three days) blunted NF-κB binding with DNA in a rat model of paraquat-induced renal injury [67]. Similarly, the administration of a beetroot extract (250 or 500 mg/kg/day for 28 days) in nephrotoxic rats reduced nuclear NF-κB protein expression and NF-κB-DNA binding activity, as well as renal inflammatory cytokines interleukin 6 (IL-6) and tumor necrosis factor α (TNF-α) [68]. Interestingly, Nrf2 is also involved in the NF-κB derived anti-inflammatory effects described for betanins. In this regard, the enhanced Nrf2-Keap1 complex dissociation induced by betanins was shown to result in greater amounts of free Keap1 in the cytosol. It must be noted that NF-κB is bonded with the KB inhibitor (IKB) kinase, constituting a complex. In order to allow NF-κB nuclear translocation to occur, the NF-κB-IKB complex needs to be dissociated by ikB kinases (IKK) [63]. IKK phosphorylates IKB by binding to the ETGE sequence, which is one of the sequences used by Keap1 to bind Nrf2. Thus, betanins induce greater availability of free Keap1, thereby enhancing IKK-Keap1 interactions (more free ETGE sequences). This, in turn, results in lower NF-κB-IKB dissociation. Consequently, betanins may reduce nuclear NF-κB translocation, and thus, prevent inflammatory gene transcription (Figure 5) [61].

Although data concerning the anti-inflammatory effects of betalains in humans are scarce, oral administration of beetroot-derived betalain-rich capsules (providing 35, 70 or 100 mg betalains/day) for 10 days has been demonstrated to be an effective approach to reducing pain and inflammation in patients suffering from osteoarthritis [69]. According to the authors, a beetroot-derived extract was shown to alleviate inflammation by decreasing levels of inflammatory cytokines like TNF-α and IL-6. It also inhibited the activity of growth regulated oncogene-alpha (GRO-alpha) and Regulated upon Activation, Normal T cell Expressed and presumably Secreted (RANTES), both considered pro-inflammatory chemokines [69].

### 3.3. Interactions and Adverse Effects of Betalains

Overall, betalain consumption (in the form of extracts or beetroot preparations) is considered to be safe. In this regard, no major adverse effects or allergic events have been reported. This may be due to their poor absorption and extensive gut metabolization [65]. Indeed, due to its safety, betanin, which is the most abundant betalain in red beetroot, is a widely used pigment in the food industry. Its usage as a red colorant has been approved by the European Food Safety Authority (EFSA) and the United Stated Food and Drug Administration (FDA) [65]. It should be noted that the consumption of products containing betalains (mainly betanin) may produce beeturia. This results in a typical coloration of urine that varies according to the quantity consumed, which is considered a consequence of the consumption of such foods and not a sign of physiological dysfunction [70].

## 4. Conclusions

The numerous studies that have been included in this comprehensive review article clearly demonstrate the health benefits, mainly regarding cardiovascular health, of the consumption of nitrate and betalains. It should be noted that when using certain formulations such as beetroot juice, both functional ingredients may well be consumed at the same time. Therefore, the health benefits attributed to nitrate and betalain consumption that have been separately described in this review article may occur concomitantly. Consequently, potential additive or synergistic effects cannot be ruled out. Although further research is still required to better understand the mechanisms underlying the beneficial effects described for nitrate and betalain consumption, the results reported to date are promising.

Additionally, since the consumption of beetroot juice is regarded as safe, future endeavors devoted to enhancing the nitrate and/or betalain content of such preparations may be expected. As such, the already well-documented health benefits and properties attributed to these compounds may be obtained/enhanced while preventing potential adverse effects that may result from the consumption of certain supplements.

## Figures and Tables

**Table 1:** Classification of different vegetables and fruits according to their nitrate content. Adapted from Moreno et al., 2015 [14].

Classification of Vegetables and Fruits According to Their NO_3_^−^ Content (mg/kg Wet Weight)				
Very low (<200 mg/kg)	Low (200–500 mg/kg)	Medium (500–1000 mg/kg)	High (1000–2500 mg/kg)	Very high (>2500 mg/kg)
Garlic Artichoke Onion Melon Pear Potato Tomato	Chikory Broccoli Cauliflower Carrot Cucumber	Turnip Cabbage	Escarole Leek Parsley Celery-root	Beetroot Spinach Lettuce Radish Celery Chard

**Table 2:** Selected studies addressing the antihypertensive effects of nitrate that have been included in this article (PICO format).

Reference	Population	Intervention	Comparison	Outcome
[24]	25 healthy, physically active adults (15 m and 10 w): - mean age 36 ± 10 years - BMI < 18.5	Consumption of a Japanese diet for 10 days (providing 18.8 mg/kg bw/day nitrate)	The controls received a non-Japanese diet (providing ≤3.7 mg/kg bw/day nitrate) for the same period	Increased plasma and saliva nitrate and nitrite levels. Significant decrease in DBP (4.5 mmHg).
[25]	30 healthy adults (15 m and 15 w) with a SBP > 120 mmHg	Single intake of 500 g BJ (containing 15 mmol nitrate/L)	Single ingestion of 500 g PL (apple juice concentrate)	Significant reduction of SBP (4–5 mmHg) in men 6 h.
[26]	18 normotensive healthy adults (18 m)	Single administration of 100, 250 or 500 g BJ diluted in mineral water (total weight of the mixture 500 g)	The controls were administered the same dose (500 g) of mineral water	SBP and DBP significantly reduced (dose dependently) over a period of 24 h.
[26]	14 normotensive healthy adults (14 m)	Single ingestion of 200 g of bread enriched with red or white beetroot (50% of the total weight)	The controls received 200 g of white bread	Significant DBP reduction over a period of 24 h.
[29]	12 old healthy adults (6 m, 6 w)	Prescription of 140 mL/d BJ (containing ≈ 9.6 mmol nitrate) during 2.5 days followed by a three-day washout period (this protocol was repeated during 6 weeks)	The controls received PL (nitrate depleted BJ) under the same conditions	A significant reduction in resting SBP, DBP and VO_2_ was found.

BJ: beetroot juice, BMI: body mass index, DBP: diastolic blood pressure, m: men, PL: placebo, SBP: systolic blood pressure, w: women.
